# Analysis of clinical effect and radiographic outcomes of Isobar TTL system for two-segment lumbar degenerative disease: a retrospective study

**DOI:** 10.1186/s12893-020-0680-8

**Published:** 2020-01-17

**Authors:** Zhi-Sheng Ji, Hua Yang, Yu-Hao Yang, Shao-Jin Li, Jian-Xian Luo, Guo-Wei Zhang, Hong-Sheng Lin

**Affiliations:** Department of Orthopedics, The First Affiliated Hospital of Jinan University, Guangzhou, 510630 China

**Keywords:** Isobar TTL, Dynamic stabilization system, Two-segment, Lumbar degenerative disease, Selective fusion

## Abstract

**Background:**

Nonfusion fixation is an effective way to treat lumbar degeneration. In the present study, we analyzed the clinical effects and radiographic outcomes of the Isobar TTL system used to treat two-segment lumbar degenerative disease.

**Method:**

Forty-one patients diagnosed with two-segment lumbar degenerative disease underwent surgical implantation of the Isobar TTL dynamic stabilization system (*n* = 20) or a rigid system (*n* = 21) from January 2013 to June 2017. The mean follow-up time was 23.6 (range 15–37) months. Clinical results were evaluated with the Oswestry Disability Index (ODI), modified Macnab criteria, and the visual analog score (VAS). Radiographic evaluations included the height of the intervertebral space and the range of motion (ROM) of the treated and adjacent segments. The intervertebral disc signal was classified using the modified Pfirrmann grading system and the University of California at Los Angeles (UCLA) system.

**Results:**

The clinical results, including the ODI and VAS, showed that there was significant improvement in the two groups after implantation and that the difference between the two groups was not significant. In addition, the clinical efficacy indicated by the modified Macnab criteria for the two groups was similar. Radiological outcomes included the height of the intervertebral space, lumbar mobility, and intervertebral disc signal. The height of the intervertebral space of the upper adjacent segment L2/3 in the rigid group was significantly lower than that in the Isobar TTL group at the last follow-up. Furthermore, the number of ROMs of the fixed-segment L3/4 in the Isobar TTL group was significantly less than that before implantation, suggesting that the fixed-segment ROMs in the Isobar TTL group were limited. In addition, the ROM of the upper adjacent segment L2/3 in the last follow-up of the rigid group increased significantly, while that of the Isobar TTL group did not change after implantation. Finally, the incidence of adjacent-segment degeneration (ASD) was significantly greater in the rigid group than in the Isobar TTL group according to the UCLA system.

**Conclusion:**

The Isobar TTL system can be clinically effective for treating two-segment lumbar degenerative disease. Compared with rigid fixation, the Isobar TTL system yielded better radiographic outcomes and maintained the mobility of the treated segments with less impact on the proximal adjacent segment.

## Background

Lower back pain (LBP) affects about 60–85% of adults at some time in their lives [1]. Lumbar disc herniation, a common degenerative disease of the spine, may lead to LBP and radicular leg pain [2]. Furthermore, the elderly may experience degeneration of intervertebral discs, which is often multisegmental and sometimes requires surgical treatment [3]. Interbody fusion is recognized as the “gold standard” treatment for lumbar degenerative diseases, but spinal fusion surgery often incurs many complications, including donor area complications, morbidity, and adjacent-segment disease [4]. A series of studies has shown that fusion surgery accelerates intervertebral disc degeneration near the area of the spinal cord fusion [5–7]. Recent data have shown this phenomenon and provided information on its occurrence, but there is a significant difference in morbidity (5–70%) [8].

Clinical experience has shown that the limitation, not the abolition, of spinal activity can relieve the symptoms of lumbocrural pain, and that it is possible to treat lumbar disease by changing the stress-transfer mode from the point of view of biomechanics [9, 10]. New spinal surgery concepts led to the development of nonfusion internal fixation systems [11]. The research and development and the clinical application of the Isobar® TTL (ATEC Spine, Inc., Carlsbad, CA, USA) nonfusion internal fixation system are based on the above-mentioned experience [12]. The Isobar TTL system is a semirigid stabilization system, first reported by Perrin in 1993, that consists of a universal pedicle screw and two dynamic rods. The dynamic rod, the key component, is a unique shock-absorption joint composed of internal superimposed titanium rings. The elastic range of motion (ROM) of the shock-absorption element is similar to the physiological motion of the spine [13]. The semirigid internal fixation device is based on a pedicle screw that can bear the load from different directions and the plane of motion of the fixed section and retain a certain ROM. In theory, internal fixation systems for treating degenerative diseases of the lumbar vertebrae can preserve the motion of the lumbar vertebrae and reduce the occurrence of adjacent-segment degeneration (ASD). With the Isobar TTL dynamic stabilization fixation system, one can perform selective fusion of spinal segments, that is, it can fix both the fusion segment and the nonfusion segment of the spine.

At present, clinical research on the use of the Isobar TTL dynamic stabilization system for the treatment of two-segment lumbar degenerative disease is still scarce. Therefore, the purpose of this retrospective study was to analyze the clinical effects and radiographic results of treating two-segment lumbar degenerative disease with the Isobar TTL dynamic stabilization system.

## Methods

### General data

From January 2013 to June 2017, 41 patients diagnosed with two-segment lumbar degenerative disease (L3/4 and L4/5) were selected according to the inclusion and exclusion criteria of the study. Twenty patients (12 males and 8 females) were included in the Isobar TTL dynamic stabilization system group and 21 patients (10 males and 11 females) were included in the rigid internal fixation group. All patients underwent regular X-ray and magnetic resonance imaging (MRI) examinations and were followed up for at least 15 months (Table 1).

**Table 1:** General data of the two study groups^a^

General data	Isobar TTL (*n* = 20)	Rigid (*n* = 21)	*P*
Age (years)	64 ± 7.78	61 ± 6.50	0.338
Sex			
Female	12 (60%)	10 (47.62%)	0.427
Male	8 (40%)	11 (52.38%)	
Follow-up (months)	22.00 ± 7.01	25.18 ± 4.75	0.227
Operation time (min)	163.64 ± 42.42	185.67 ± 27.80	0.138
Intraoperative blood loss (mL)	245.45 ± 145.70	445.00 ± 305.00	0.067
Hospital stay (days)	20 ± 4.22	18.6 ± 1.92	0.187

^a^Data are presented as mean ± standard deviation

*P* values are based on the *t* test; *P* > 0.05 for the Isobar TTL group compared with the rigid fixation group

### Inclusion, exclusion, and fusion criteria

The inclusion criteria were as follows: (a) spinal surgery patient at the First Affiliated Hospital of Jinan University from January 2013 to June 2017; (b) diagnosis of lumbar degenerative disease in two consecutive segments (L3/4 and L4/5) for which conservative treatment for more than 3 months was ineffective; (c) good compliance with and informed consent given to the surgical program and active cooperation with the treatment of the clinical researchers; (d) follow-up for at least for 15 months.

The exclusion criteria were as follows: (a) severe scoliosis or sagittal or coronal imbalance; (b) poor physical condition, unable to tolerate surgery, or had surgical contraindications; (c) incomplete medical records or imaging data.

Fusion criteria included (a) severe disc degeneration; (b) intervertebral instability; (c) significant lumbar degenerative scoliosis, kyphosis, or spondylolisthesis; (d) bilateral facetectomy > 1/3–1/2, excision of more than 50% of the pars interarticularis, bilateral discectomy, and partial facetectomy.

### Operative methods and postoperative management

In this study, all patients were operated on while in the prone position by the same surgical team. The surgeons used the midline approach where the muscles around the vertebrae are peeled off to expose the corresponding spinal segments. A C-arm X-ray machine guided the safe placement of the pedicle screws. The scope of the decompression of the spinal canal depended on the condition of the patient. For decompression, the nucleus pulposus is cleared, the nerve root is thoroughly decompressed, and the fusion cage, filled with autologous bone, is inserted into the intervertebral disc. For the Isobar TTL patients, the selection of the fusion segments was based on the degree of disc degeneration and the severity of the disease, whereas for the rigid group patients, all surgical segments were fused. In the rigid group, rigid titanium rods were placed at the end of the nail, and in the Isobar TTL group, L3/4 was selected for nonfusion.

After surgery, the patients were given broad-spectrum antibiotics for 24 h, and the drainage tube was removed when the drainage volume was < 50 mL over 24 h. After discharge from the hospital, patients were ordered to undergo regular reexaminations, wear waist circumference protection for 3 months, and not do excessive weight-bearing activities for 6 months.

### Clinical and radiological outcomes

Clinical and radiological data were obtained before surgery and at the final follow-up. VAS and ODI were used to evaluate the LBP and quality of life of each patient. The clinical efficacy of the surgery for the two groups was evaluated using the Greenough judgment standard of the clinical curative effect.

Radiological outcomes were evaluated using the following: (a) the fusion rate, which was determined using the judgment standard of bone fusion established by Suk [14]; (b) the segmental ROM, which was calculated as the angle between the inferior surface of the upper vertebrae and the superior surface of the lower vertebrae on the lateral lumbar flexion-extension X-ray taken with the patient standing; (c) the ventral intervertebral space height; (d) lumbar MRI taken at the final follow-up to evaluate changes in the height of the adjacent degenerative intervertebral discs (L2/3) and in the signals of the intervertebral discs (disc degeneration was graded on T2-weighted sagittal and axial MRI scans according to the modified method described by Pfirrmann); and (e) the University of California at Los Angeles (UCLA) grading scale system, which classifies the degree of intervertebral space degeneration evaluated from X-rays. In this study, two experienced spinal surgeons conducted three independent assessments of radiographs.

### Statistical assessment

The clinical data and imaging measurements of the 41 patients were analyzed using SPSS v19.0 software (IBM, Armonk, NY, USA). Measurement data are presented as mean ± standard deviation (SD). Enumeration data were evaluated with the χ^2^ test and categorical data were compared using the Wilcoxon signed rank test; *P* < 0.05 was considered statistically significant.

## Results

### Patient baseline characteristics

All patients were followed up for 15–37 months: the Isobar TTL group for an average of 22.00 months and the rigid group for an average of 25.18 months. Operation time for the Isobar TTL group ranged from 125 to 199 min (average time = 163.64 min) and that for the rigid group ranged from 145 to 222 min (average time = 185.67 min). Intraoperative blood loss was 245 mL for the Isobar TTL group and 300–1300 mL for the rigid group. There was no statistically significant difference between the two groups with respect to age, bleeding volume during surgery, follow-up period, total hospital stay, and operation time (Table 1).

### Clinical efficacy

The ODI of the Isobar TTL group of patients was 81.84 ± 6.63 before surgery and 30.15 ± 4.38 at the last follow-up, a significant improvement from the preoperative value. The ODI of the rigid group of patients on admission was 82.21 ± 5.86 and 28.06 ± 5.39 at the last follow-up, a 65.87% improvement from the preoperative value. The VAS for the Isobar TTL group was 6.82 ± 1.77 points before surgery and 2.75 ± 0.86 points at the last follow-up. The VAS for the rigid group was 6.70 ± 1.51 points before surgery and 2.58 ± 0.86 points at the last follow-up. The ODI and VAS values for the Isobar TTL group and the rigid group at the last follow-up were not significantly different, but the *P* values of ODI and VAS of the Isobar TTL group were < 0.05 before and after surgery. However, there was no significant difference in the improvement of ODI and VAS between the two groups (Table 2).

**Table 2:** ODI and VAS values^a^

	Isobar TTL (*n* = 20)		Rigid (*n* = 21)	
	ODI	VAS	ODI	VAS
Before surgery	81.84 ± 6.63	6.82 ± 1.77	82.21 ± 5.86	6.70 ± 1.51
After surgery	30.15 ± 4.38	2.75 ± 0.86	28.06 ± 5.39	2.58 ± 0.86
*P*	0.000	0.000	0.000	0.000
*P* ′		ODI (0.182)		VAS (0.530)

^a^Data are presented as mean ± standard deviation

*P* values are based on the paired *t* test. *P* is for postoperative values compared with preoperative values; *P* < 0.05. *P′* is for the Isobar TTL group compared with the rigid group; *P* > 0.05 indicates a statistically significant difference

The Greenough judgment standard of the clinical curative effect showed that the Isobar TTL dynamic group at the last follow-up had an excellent-good rate of 85.0%, while the rigid group had an excellent-good rate of 71.4%. However, there was no significant difference between the Isobar TTL group and the rigid group (*P* > 0.05) (Table 3).

**Table 3:** Clinical assessment of the Greenough judgment of the clinical effect

Group	*n*	Excellent	Good	Fair	Poor	Excellent-Good rate	*P*
Isobar TTL	20	4	13	3	0	85.00%	1
Rigid	21	2	13	5	1	71.40%	

*P* values are based on the χ^2^ test. *P* < 0.05 indicates a statistically significant difference

### Radiological outcomes of fusion rate

At the last follow-up, there were 20 fusion segments in the Isobar TTL group, where 19 were judged as strong fusion and 1 as possible fusion, for a fusion rate of 95.00%. There were 42 fusion segments in the rigid fixation group, where 40 were judged as strong fusion and 2 as possible fusion, for a fusion rate of 97.30%. There was no statistical difference in fusion rate between the two groups (*P* > 0.05) (Table 4).

**Table 4:** Fusion rate of the two groups

Grading	Isobar TTL (*n* = 20)	Rigid (*n* = 42)	*P*
Fusion	19	40	
Possible fusion	1	2	
Nonfusion	0	0	
Fusion rate (%)	95.00	95.20	1.000

*P* values are based on the χ^2^ test, *P* < 0.05 indicates a statistically significant difference

### Radiological outcomes of lumbar mobility and height of intervertebral space

**Fig. 1:**
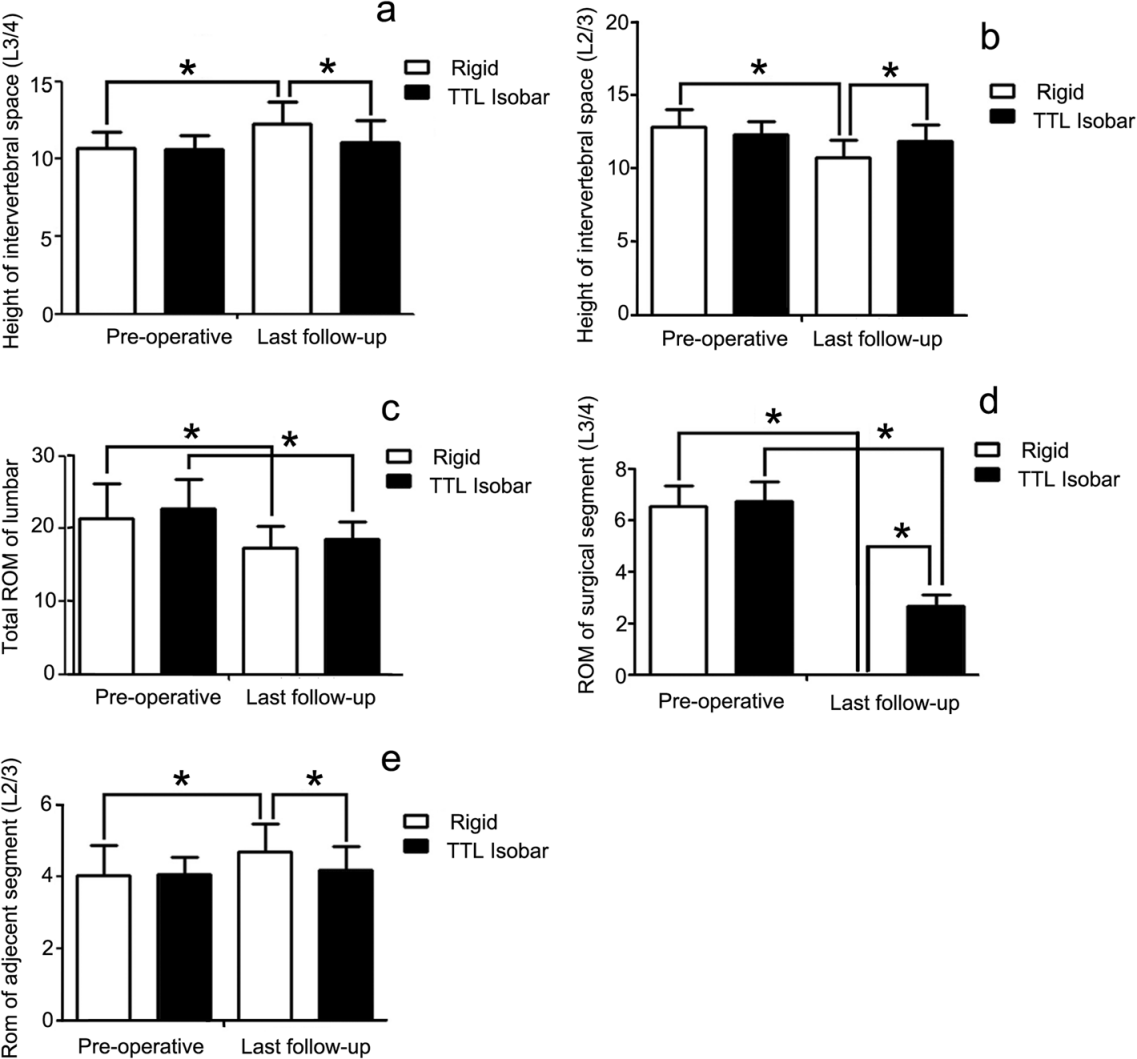
Comparison of the effects of the Isobar TTL dynamic stabilization system and the rigid fixation system on lumbar mobility and height of intervertebral space. **a** The intervertebral space height of L3/4, the surgical segment, in the Isobar TTL and rigid groups before surgery and at the last follow-up. **b** The intervertebral space height of L2/3, the upper adjacent segment, in the Isobar TTL and rigid groups before surgery and at the last follow-up. **c** ROMs of the nonfusion fixed segment before surgery and at the last follow-up. **d** The ROM of the surgical segment L3/4 before surgery and at the last follow-up in the two groups. **e** The ROM of upper adjacent segment L2/3 before surgery and at the last follow-up in the two groups. * means *P* < 0.05

**Fig. 2:**
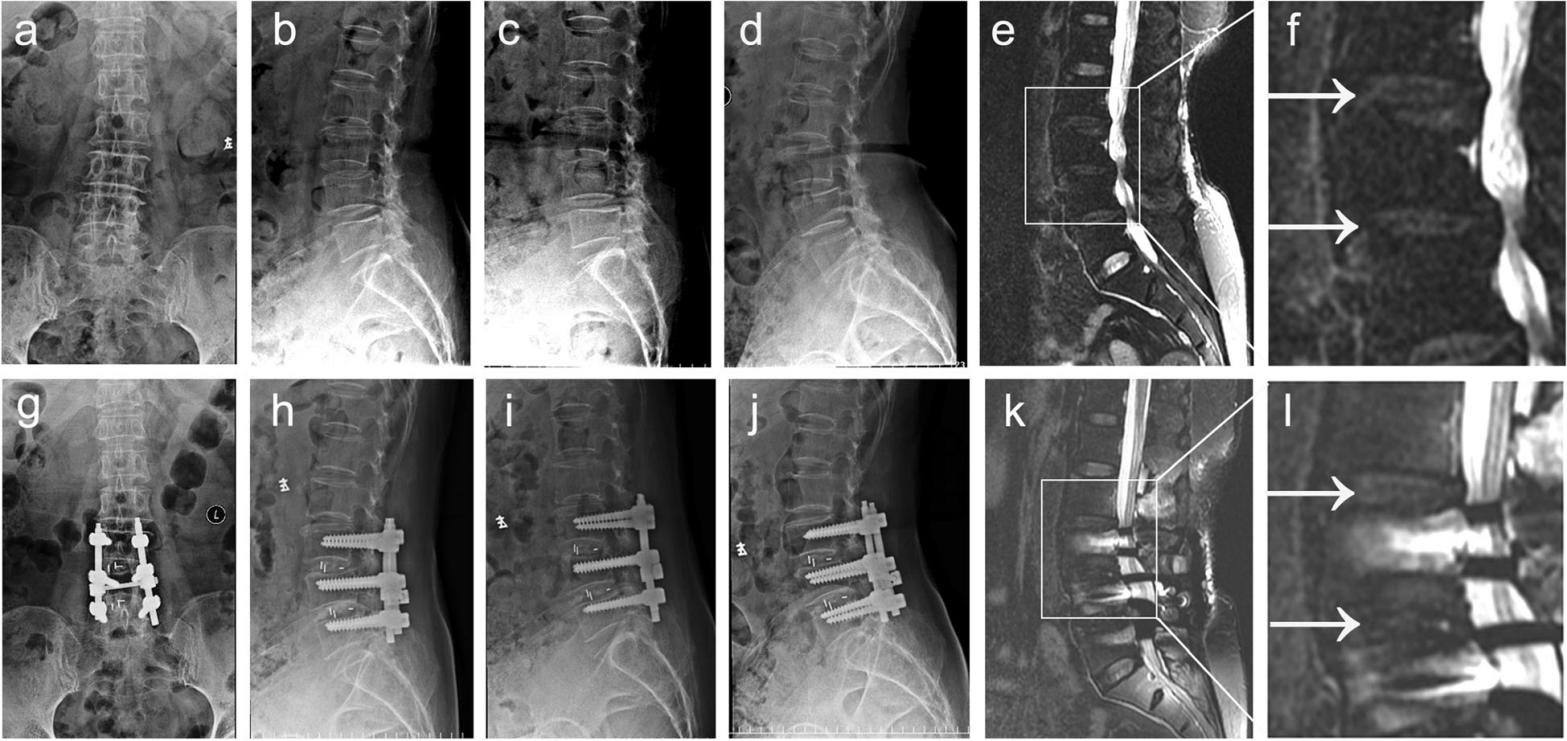
Typical case in the Isobar TTL group: A old patient with two-segment degenerative lumbar disease (L3/4 and L4/5). **a-d** X-ray and **e** and **f** MRI images of the patient before surgery; **g-i** X-ray and **j-l** images of the patient at the last follow-up

**Fig. 3:**
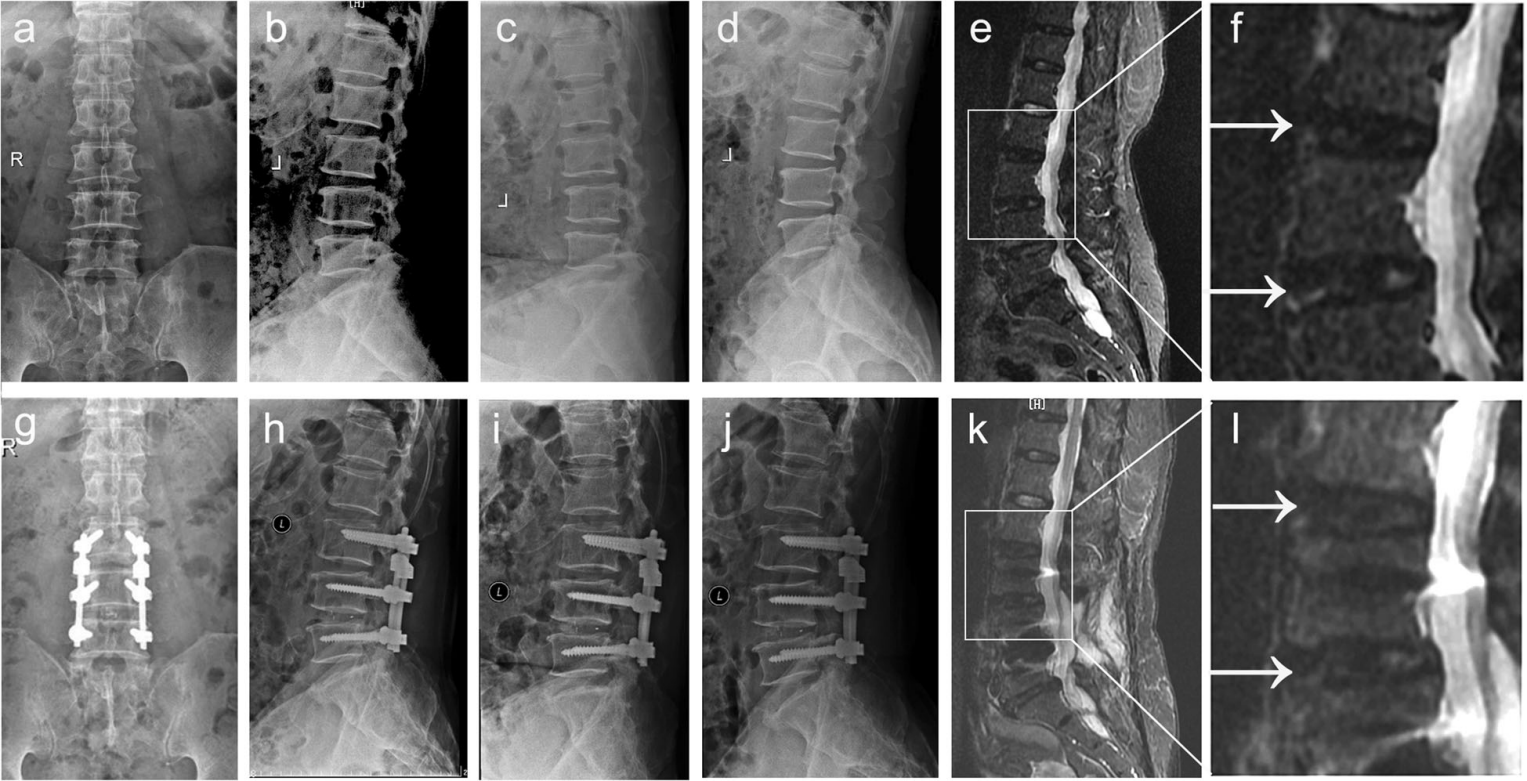
Typical case in the rigid group: A old patient with two-segment degenerative lumbar disease (L3/4 and L4/5) underwent L3/4 and L4/5 decompression and rigid fixation. **a-d** X-ray and **e** and **f** MRI images of the patient before surgery; **g-j** X-ray and **k** and **l** MRI images of the patient at the last follow-up

The preoperative and postoperative heights of the intervertebral space in the Isobar TTL and rigid groups are shown in Fig. 1 and two groups' typical case are shown in Figs. 2 and 3. The preoperative heights of the L2/3 and L3/4 intervertebral spaces for the two groups were similar (*P* > 0.05). In addition, the postoperative height of the L3/4 intervertebral space in the rigid group at the last follow-up was better than that in the Isobar TTL group, which shows that the intervertebral fusion cage helps the recovery of the intervertebral height. However, the height of the intervertebral space between the upper adjacent segment L2/3 in the rigid group was less than that in the Isobar TTL group at the last follow-up (*P* < 0.05), indicating that the Isobar TTL probably slows down the degeneration of adjacent segments to a certain extent (Fig. 1b).

The preoperative and postoperative radiological parameters, including total lumbar mobility and L2/3 and L3/4 ROM, in both groups are shown in Fig. 1. The total lumbar ROM of the two groups at the last follow-up was significantly less than that before surgery (*P* < 0.05). There was no statistical difference in the total lumbar ROM between the two groups at the last follow-up (*P* > 0.05) (Fig. 1c).

The ROMs of the fixed segment L3/4 in the Isobar TTL group at the last follow-up were significantly less than those before surgery (*P* < 0.05), suggesting that the ROMs of the fixed segment in the Isobar TTL group were limited, but some spinal motion was retained. The fixed-segment L3/4 in the rigid group was immobile because it was fused (Fig. 1d).

The ROM of upper adjacent segment L2/3 in the last follow-up of the rigid group increased significantly (*P* < 0.05), while the ROM of L2/3 in the Isobar TTL group did not change from before surgery to the last follow-up. In addition, the ROM of the upper adjacent segments in the rigid group had increased significantly compared with that in the Isobar TTL group at the last follow-up (*P* < 0.05), indicating that the Isobar TTL was better than the rigid fixation system in retaining a certain ROM of the lumbar spine.

### Radiological outcomes of degeneration in adjacent segments

According to the UCLA system, the incidence of ASD was 5.0% in the Isobar TTL group and 19.0% in the rigid group. Thus, ASD was significantly slower in the Isobar TTL group than in the rigid group (*P* < 0.05) (Table 5).

**Table 5:** UCLA system evaluation of intervertebral space (*N* = 41)

Segment	Isobar TTL (*n* = 20)	Rigid (*n* = 21)
L2/3	1 (5.0%)	4 (19.0%)
L3/4	1 (5.0%)	0 (0.0%)

*P* values are based on the χ^2^ test. *P* < 0.05 indicates a statistically significant difference.

## Discussion

The goal of the Isobar TTL dynamic stabilization system is to maintain spinal stability while partially preserving the mobility of the surgical segment, thus avoiding ASD. Traditional rigid fusion changes the transmission mode of spinal mechanics, increases the stress on adjacent segments, and makes it easier for ASD to occur. The use of the Isobar TTL dynamic stabilization system has many advantages in the treatment of two-segment lumbar degenerative diseases. The pedicle screw-based semirigid internal fixation device reduces the pressure on the intervertebral joint, protects the interbody joint of the nonfusion segment [15], bears the load on the fixed segment from different directions and motion planes, disperses the axial load and the flexion and extension shear forces on the intervertebral disc, and stabilizes the lumbar vertebrae while retaining a certain degree of lumbar motion. In addition, it disperses the stress on adjacent segments thus reducing or delaying ASD [16]. Therefore, the Isobar TTL dynamic stabilization system, rather than rigid fusion, is particularly suitable for treating two-segment lumbar degenerative diseases with relatively mild symptoms of segmental motion fixation [17]. Thus, in theory, the Isobar TTL dynamic stabilization system has more advantages than a rigid fixation system.

In our study, both the Isobar TTL dynamic stabilization system and the rigid fixation system for the treatment of two-segment lumbar degenerative disease yielded a good clinical effect. The VAS score and ODI for the two groups were significantly improved postoperatively and at the final follow-up. However, the difference between the VAS and ODI values for the Isobar TTL group and the rigid group was not significant. In addition, using the Greenough judgment standard to evaluate clinical efficacy, we found that the excellent-good rate for the Isobar TTL group was 85.0% and that for the rigid group was 71.4%, but the difference was not statistically significant (*P* > 0.05). Therefore, the outcome of the Isobar TTL group was similar to that of the traditional internal fixation system group. This suggests that the Isobar TTL dynamic stabilization system has a reliable curative effect in treating multilevel lumbar degenerative disease.

The Isobar TTL dynamic stabilization system retains some of the motion of the surgical segment based on the stability of the lumbar spine. In addition, it preserves the ROM of the lumbar spine. Some studies have reported that the intervertebral space is restored soon after surgery but will gradually decrease over the long term [18, 19]. A possible reason for the restoration is that the intervertebral space is properly elevated by lumbar surgery. In this study, the intervertebral space in the Isobar TTL group after surgery was slightly higher than before surgery, providing the possibility of maintaining a better and stable level of intervertebral space after internal fixation. However, with the occurrence of intervertebral space degeneration, the height of the intervertebral space gradually decreased. In addition, the height of the intervertebral space of the upper adjacent segment in the rigid group decreased more rapidly than in the Isobar TTL group, indicating that the Isobar TTL dynamic stabilization system slows down ASD.

The Isobar TTL dynamic stabilization system can restore the lumbar disc height of the surgical segment and maintain the lumbar structure, as opposed to lumbar interbody fusion. Furthermore, it can preserve the ROM of the surgically corrected segment, thus reducing the stress load on the adjacent segments and compensating the ROM [20, 21]. Other studies have also supported the view that the dynamic stabilization system can reduce ASD [22–24]. In this study, the total lumbar ROM of the Isobar TTL group at the last follow-up was significantly greater than that of the rigid group, indicating that Isobar TTL helps retain a certain degree of the total lumbar ROM. The change in the ROM of the upper adjacent segment in the Isobar TTL group was less than that in the rigid group. The Isobar TTL dynamic stabilization system had little effect on the relative activity of adjacent segments. The postoperative mobility of the fixed segment of the Isobar TTL group was significantly lower than that before surgery, but partial mobility was retained. The Isobar TTL system has a stabilizing effect on the surgical segment of the lumbar vertebrae, but the effect is different than the lack of intervertebral motion seen in a rigid fusion segment.

The idea behind the Isobar TTL dynamic stabilization system is to preserve the dynamic changes made to the surgical segment, reduce the pressure on the intervertebral joint, and reduce its compensatory activity. In addition, dynamic stabilization may indirectly reduce ASD [25]. The improved Pfirrmann classification system used in this study showed that two adjacent segments in the Isobar TTL group (10%) and three adjacent segments in the rigid fixation group (23.8%) showed degeneration (date not showed). However, the UCLA system found that one adjacent segment in the Isobar TTL group (5%) and four adjacent segments in the rigid group (19%) showed degeneration. Both evaluation systems showed that ASD in the rigid group was more obvious than that in the Isobar TTL group, indicating that the Isobar TTL dynamic system slows down ASD to a certain extent, in accordance with the design concept of the Isobar TTL dynamic stability system [26].

In general, treatment of degenerative spinal disease must maintain the stability of the spine after surgery, decrease further degeneration of the lumbar vertebrae, retain the normal ROM of the treated segment, and minimize the complications caused by internal fixation in the long term [27, 28]. The use of the Isobar TTL posterior internal fixation system to treat two-segment lumbar degenerative disease provides a new way to clinically preserve spinal joint motility. The results of this retrospective study suggest that the Isobar TTL is no worse than the traditional method for treating two-segment lumbar degenerative disease, especially with respect to preserving the degree of motion of the spinal motor joint. Further randomized, controlled, prospective, multicenter clinical studies are needed to provide more evidence of the therapeutic effect of the system in treating lumbar degenerative diseases.

## Conclusions

Compared with a rigid fixation system for the treatment of two-segment lumbar degenerative disease, the Isobar TTL dynamic stabilization system obtained good treatment results, better radiographic outcomes, and maintenance of the mobility of the stabilized segments with less of an effect on the proximal adjacent segment. By preserving segmental motion and intervertebral height and maintaining lumbar lordosis, the Isobar TTL may reduce the incidence of ASD.

## Data Availability

The datasets used and analyzed in this study are available from the correspondence author upon reasonable request.

## Abbreviations

ASD: Adjacent segment degeneration
LBP: Low back pain
MRI: Magnetic resonance imaging
ODI: Oswestry Disability Index
ROM: Range of motion
UCLA: University of California at Los Angeles
VAS: Visual analog score
